# Incidence of antiretroviral therapy regimen modification and associated factors among People Living with HIV in Beijing, China

**DOI:** 10.1371/journal.pgph.0005319

**Published:** 2025-11-03

**Authors:** Yuanqi Mi, Xiaofei Wang, Yuhong Zeng, Yang Guo, Peicheng Wang, Feng Cheng, Mengge Zhou

**Affiliations:** 1 Department of Epidemiology, Bloomberg School of Public Health, Johns Hopkins University, Baltimore, Maryland, United States of America; 2 School of International Organizations, Beijing Foreign Studies University, Beijing, China; 3 Clinical Research Center, Sichuan Provincial People’s Hospital, University of Electronic Science and Technology of China, Chengdu, China; 4 Department of Epidemiology and Statistics, School of Public Health, Hebei Key Laboratory of Environment and Human Health, Hebei Medical University, Shijiazhuang, China; 5 Vanke School of Public Health, Tsinghua University, Beijing, China; 6 Department of Epidemiology and Biostatistics, Institute of Basic Medical Sciences Chinese Academy of Medical Sciences, School of Basic Medicine Peking Union Medical College, Beijing, China; Kiruddu National Referral Hospital, UGANDA

## Abstract

Durability of the initially prescribed antiretroviral therapy (ART) regimen is critical for long-term virologic suppression among people living with human immunodeficiency virus (HIV). However, data on the incidence of regimen modification and its associated factors remain limited in China. We aim to quantify the incidence of initially prescribed ART modification and identify associated baseline factors in China. Treatment-naïve adults (≥18 years) who initiated ART with complete regimen records and have documented follow-up information in Beijing, China, from 2010 to 2020 were included. The primary outcome was initially prescribed ART regimen modification. A multivariable Cox proportional hazards model was applied to evaluate factors associated with modification risk. Of 18,911 participants included, 3,725 (19.7%) participants experienced ART modification over 472,565 person-months of follow-up (PMFU), a rate of 7.9 per 1,000 PMFU. The median follow-up was 32 (IQR 13–36) months. Modification rates peaked in months 0–6: TDF + AZT + NVP and LPV/r + 3TC + AZT exhibited the highest 6-month modification rates (59.4 and 57.7 per 1,000 PMFU, respectively), whereas TDF + 3TC + EFV, the most prescribed regimen, had the lowest early switch rate (8.8 per 1,000 PMFU). In multivariable analysis, baseline white blood cell (WBC) < 4.0 × 10⁹/L and WHO stage II–IV were associated with higher modification risk; missing baseline records of WBC, hepatitis B virus or hepatitis C virus coinfection, and later calendar year of ART initiation were associated with lower modification risk; compared to EFV + 3TC + TDF, LPV/r + 3TC + AZT had the highest modification risk. TDF + 3TC + EFV was the predominant initially prescribed regimen with the highest durability, while LPV/r + 3TC + AZT had the highest modification rate. These findings underscore the need for early ART initiation, comprehensive pretreatment screening, and enhanced early monitoring—especially during the first six months—to optimize regimen selection and minimize unnecessary modification.

## Introduction

Human immunodeficiency virus (HIV) remains a significant global public health issue, with an estimated 39.9 million people living with HIV (PLWH) by 2023 worldwide and 1.26 million in China [1]. Since 2016, the World Health Organization (WHO) has recommended universal test-and-treat (UTT), whereby all PLWH initiate antiretroviral therapy (ART) regardless of CD4 cell count or clinical stage [2]. Since 2003, China implemented the National Free Antiretroviral Treatment Program (NFATP), which has widely expanded ART access [3]. The operational workflow, from key population screening, confirmatory testing, registration in the national case-management system, to linkage with ART clinics, was structurally delayed before 2016 in China by CD4-based ART eligibility assessment [4]. China’s adoption of UTT in 2016 removed these restrictions and further promoted ART coverage [5,6].

The expanded coverage of ART has transformed HIV from a fatal disease into a manageable chronic condition [7], expediting the aging epidemic trend of HIV [8]. To select the optimal regimen, China’s ART guidelines have been periodically updated since 2010 and incorporated WHO’s evolving regimen recommendations—while also taking into account domestic factors such as drug approval timelines, local safety and efficacy data, and cost considerations [9]. Consequently, people on lifelong therapy will inevitably cycle through multiple ART regimens over time [10]. However, the durability of the initially prescribed ART regimen is crucial for its long-term efficacy on PLWH [11]. Inappropriate modification of ART was found to be associated with the development of viral cross-resistance to a specific regimen [12] and increased mortality [13] among PLWH. Additionally, subsequent regimens have progressively shorter durability and are often more expensive [14].

Evidence on associated factors of initially prescribed ART regimen modification in China remains limited and lacks exploration on patients’ baseline clinical comorbidities [11]. Previous studies have shown that toxicity and side-effects of ART are the most common reasons for treatment modification, often influenced by demographic characteristics and drug-drug interactions [11,12,15–18]. Understanding these factors is critical for optimizing treatment durability, minimizing resistance, and improving long-term outcomes among PLWH.

To examine regimen modification in Beijing, China, the objective of this study is to describe incidence, and associated factors of initially prescribed ART modifications.

## Materials and methods

### Ethics approval

The study was approved by the Ethics Committee of Beijing Center for Disease Control and Prevention on May 17, 2018 with approval identification assigned: No. 2018 [4]. Confidentiality was protected for individual information processing. The data used in our study were de-identified clinical records; therefore, informed consent was not required.

### Study design and population

**Fig 1:**
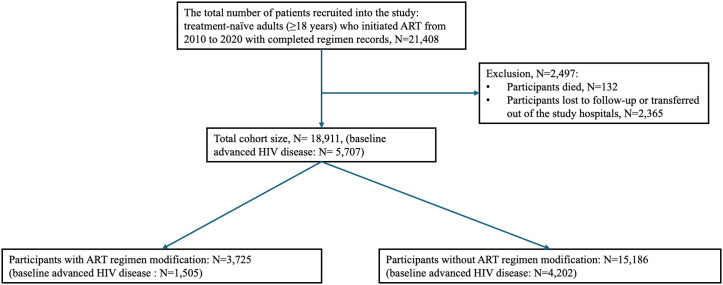
Flow chart: Inclusion and exclusion criteria.

This retrospective cohort study used data collected from a surveillance system that tracked clinical information on PLWH receiving ART and long-term follow-up care at the STD/AIDS Prevention and Treatment Institute of the Beijing Center for Disease Prevention and Control (CDC) in China [19]. Clinical data were reported by clinicians from four designated HIV treatment hospitals in Beijing, including Peking Union Medical College Hospital, Beijing You’an Hospital, Beijing Ditan Hospital and 302 Military Hospital of China. The baseline data included 21,408 treatment-naïve PLWH aged 18 or above who initiated ART at one of these hospitals from 2010 to 2020 with complete records of ART regimen. Regular clinical follow-up was conducted at least once per 6 months. Patients who died (n = 132), were lost to regular follow-up, or were transferred out of the study hospitals (n = 2,365) were excluded from the study, accounting for 11.7% (2,497/21,408) of the initial cohort (Fig 1). Baseline sociodemographic, clinical characteristics, as well as laboratory measurements of ART-naïve patients were assessed. All Laboratory measures were tested at the centralized hospital laboratory in each hospital in accordance with standardized operating procedures in China. Clinical characteristics were collected from patients’ electronic health records reported by clinicians according to standardized reporting requirements for the national surveillance. Data for this study were accessed on January 4, 2022. The data has no time-limited access restrictions.

### Measurements

ART modification was defined as (i) replacement of one or more nucleoside reverse transcriptase inhibitor (NRTIs) or non-nucleoside reverse transcriptase inhibitor (NNRTIs) within the first-line regimen by another drug of the same class, or (ii) a switch from a first-line to a second-line regimen. Since lamivudine (3TC) and emtricitabine (FTC) are therapeutically interchangeable, a change between these two drugs was not regarded as a modification [20].

Regimen data assessed included initially prescribed ART combination, subsequent modifications, and corresponding dates. Marital status included single, married or cohabiting, divorced or separated, or widowed. Infection routes included homosexual transmission, heterosexual transmission, or other transmission (including non-sexual transmission). Body mass index (BMI) was calculated as weight in kilograms divided by height in meters squared. The laboratory testing included CD4 count, HIV viral load, hepatitis B virus (HBV), and hepatitis C virus (HCV) at ART initiation. Opportunistic infection was defined as a patient with at least one opportunistic infection within three months before ART initiation. HBV infection was defined as having a positive hepatitis B surface antigen (HBsAg) result on the baseline laboratory test. HCV infection was defined as the presence of anti-HCV antibody and a confirmatory nucleic acid test that proved HCV RNA was detectable. Clinical manifestation of HIV was defined as a patient with one of the following symptoms at the initiation of ART: fever, cough, expectoration, dyspnea, chest pain, night sweats, diarrhea, nausea, projectile vomiting, headache, decreased vision, blurred vision, rash, or swollen lymph nodes. WHO HIV clinical staging was judged by clinicians. Tuberculosis was defined as a patient having self-reported tuberculosis within one year before ART initiation. Advanced HIV disease was defined as having the laboratory testing result of CD4 cell count <200 cells/mm^3^ or were clinical defined as WHO HIV stage 3 or 4 at ART initiation [21]. Anemia was defined as hemoglobin (HB) ≤130 g/L for males and ≤120 g/L for females. The aspartate aminotransferase (AST) to Aspartate Aminotransferase-to-Platelet Ratio Index (APRI) was calculated by dividing the AST level by the upper limit of normal for the laboratory (i.e., the AST elevation), then dividing this value by the platelet count (expressed as the number of platelets per mm³ divided by 1000 to convert to 10⁹/L), and finally multiplying the result by 100 [17]. APRI was categorized using a validated cutoff of 0.5, and APRI ≥0.5 was designated as liver fibrosis [22]. The estimated glomerular filtration rate (eGFR) was calculated by an equation developed by the Chronic Kidney Disease Epidemiology Collaboration [23]. Delayed ART initiation was defined as a period of more than 30 days between HIV diagnosis and ART initiation. Since most PLWH have incomplete records for different clinical characteristics, we created a separate category as “missing” for variables with missingness to measure the lack of clinical monitoring at ART initiation of PLWH.

### Statistical analysis

Normality of the continuous variables, including BMI, age, fasting blood glucose (FBG), eGFR, CD4, alanine transaminase (ALT), white blood cell (WBC), triglyceride (TG), total cholesterol (TC), and total bilirubin (Tbil) were tested using *Shapiro–Wilk* and *Kolmogorov–Smirnov* tests [24]. Continuous variables with normal distributions were reported as means (standard deviations), and differences between groups were compared using *t*-tests; continuous variables with skewed distributions were reported as medians (interquartile ranges [IQR]), and differences between groups were compared using *Mann-Whitney U* tests. All categorical variables were presented as the number (percentage) and compared using the *chi-square* test. Percentages were used to describe baseline information of patients. The incidence rate of ART modification was calculated by dividing the number of patients with initial first-line ART modification by the total number of person-month of follow-up (PMFU). Kaplan–Meier estimates and log-rank tests were carried out to compare the cumulative incidence of ART modification between different initially prescribed ART regimens, backbones, and anchors. The Cox proportional hazards model, a semi-parametric regression method, was used to model time to first ART regimen modification accounting for right censoring and unequal follow-up and analyze the factors associated with ART regimen modification. This model estimates hazard ratios (HRs) and 95% confidence intervals (CIs) while assuming that covariate effects are multiplicative and remain constant over follow-up (proportional hazards assumption) [25]. The proportional hazards assumption was assessed graphically by inspecting the log-log survival curves for parallelism across covariate strata. The following baseline characteristics were considered as candidate variables in a multivariable Cox proportional hazards model of ART modification, including sex assigned at birth, age, BMI, WBC, WHO stage, tuberculosis, HBV or HCV seropositive, anemia, FBG, eGFR, TC, ALT, Tbil, delayed ART initiation, ART regimen type, and year of ART initiation. Except for age, all other candidate variables were treated as categorical in the model. We filtered these candidate variables through a backward stepwise multivariable Cox proportional hazards regression model, with variables being included in the model if it resulted in an improvement in the model fit. Statistical analyses were performed using IBM’s Statistical Package for the Social Sciences (SPSS) version 23.0 for Windows (IBM Corp., Armonk, NY, USA) and R software, version 4.2.3 (R Foundation for Statistical Computing, Vienna, Austria, 2023) [26]. Two-tailed *p*-values of less than 0.05 were considered statistically significant.

## Results

### Baseline sociodemographic and clinical characteristics

A total of 18,911 PLWH were included in the analysis. The median age at initiation was 31 (IQR 26–39) years, and only 4.3% were female (Table 1). The median BMI overall was 22.0 (IQR 20.1–24.1) kg/m² with 1,359 (7.2%) underweight (<18.5 kg/m^2^). The majority were single (68.8%) and acquired HIV through homosexual transmission (82.7%). Opportunistic infections and HIV-related clinical manifestations were each present in fewer than 5% of patients at baseline (4.4% and 4.6%, respectively), and 78.4% were on WHO stage I. Nearly half (48.4%) experienced delayed ART initiation (>30 days after diagnosis). Coinfection with HBV or HCV was observed in 5.5%, anemia in 10.9%, and liver fibrosis in 11.7%.

### Differences in baseline characteristics by treatment modification

Overall, a total of 3,725 (19.7%) PLWH experienced ART regimen modification over 472,565 PMFU, a rate of 7.9 per 1,000 PMFU. The overall median follow-up time was 32 (IQR 13–36) months. When stratified by ART regimen modification, several characteristics diverged markedly from the overall cohort. Participants with regimen modification were more likely to be female (6.0% vs 3.9% without regimen modification), have baseline advanced HIV disease (41.5% vs 28.7% without regimen modification), have delayed ART initiation (57.2% vs. 46.3% without regimen modification), and have tuberculosis (1.6% vs. 0.6% without regimen modification). Participants with regimen modification were less likely to be HBV or HCV seropositive (3.7% vs. 5.9% without regimen modification) (Table 1).

### Trends in initially prescribed ART regimens

**Fig 2:**
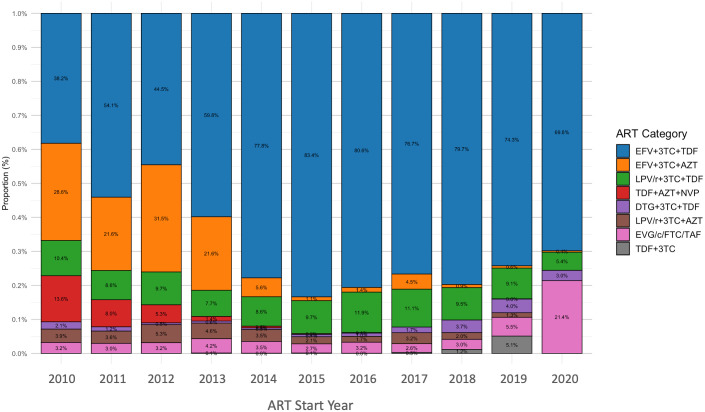
Trend of initially prescribed ART regimens for each year.

The trend of initially prescribed ART regimens from 2010 to 2020 was presented in Fig 2. In 2010, EFV (Efavirenz)+3TC + TDF (Tenofovir Disoproxil Fumarate) comprised 38.2% of initially prescribed regimens, followed by EFV + 3TC + AZT (Zidovudine) (28.6%) and LPV/r (Lopinavir boosted with Ritonavir)-based regimens (LPV/r + 3TC + TDF and LPV/r + 3TC + AZT) (24.0%). The proportion of EFV + 3TC + TDF rapidly reached 77.8% by 2014 and peaked at 83.4% in 2015, while EFV + 3TC + AZT fell below 6% and LPV/r + 3TC + TDF stabilized around 8–12%. From 2016 to 2018, EFV + 3TC + TDF remained above 75%. In 2019, the proportion of INSTI-based options increased (EVG/c (Elvitegravir boosted with Cobicistat) + FTC + TAF (Tenofovir Alafenamide): 5.1%, DTG + 3TC + TDF: 5.5%). By 2020, DTG (Dolutegravir) + 3TC + TDF had risen to 21.4%, EVG/c + FTC + TAF to 3.0%, and EFV + 3TC + TDF correspondingly declined to 69.8% Table 1.

**Table 1:** Characteristics of patients at ART initiation.

	Total Patients (N = 18911)	Patients without ART regimen modification (N = 15186)	Patients with ART regimen modification (N = 3725)	P-value
Female, n (%)	815 (4.3)	591 (3.9)	224 (6.0)	<0.001
Age years, median (IQR)	31 (26, 39)	31 (26, 38)	31 (26, 40)	0.014
Age, years, n (%)				0.028
≥18 to <30	8307 (43.9)	6731 (44.3)	1576 (42.3)	
≥30 to <45	7736 (40.9)	6202 (40.8)	1534 (41.2)	
≥45 to <60	2437 (12.9)	1921 (12.6)	516 (13.9)	
≥60	431 (2.3)	332 (2.2)	99 (2.7)	
BMI, kg/m^2^, median (IQR)	22.0 (20.1, 24.1)	22.0 (20.1, 24.1)	21.6 (19.9, 23.7)	<0.001
BMI, kg/m^2^, n (%)				<0.001
<18.5	1359 (7.2)	1064 (7.0)	295 (7.9)	
≥18.5 to <24.9	10539 (55.7)	8551 (56.3)	1988 (53.4)	
≥25	2450 (13.0)	2053 (13.5)	397 (10.7)	
missing	4563 (24.1)	3518 (23.2)	1045 (28.1)	
Marital status, n (%)				<0.001
Single	13015 (68.8)	10580 (69.7)	2435 (65.4)	
Married/cohabitating	4651 (24.6)	3608 (23.8)	1043 (28.0)	
Divorced/separated/widowed	1062 (5.6)	853 (5.6)	209 (5.6)	
missing	183 (1.0)	145 (1.0)	38 (1.0)	
Infection route, n (%)				<0.001
Homosexual	15647 (82.7)	12710 (83.7)	2937 (78.8)	
Heterosexual	1847 (9.8)	1409 (9.3)	438 (11.8)	
Other	211 (1.1)	136 (0.9)	75 (2.0)	
Missing	1206 (6.4)	931 (6.1)	275 (7.4)	
Opportunistic infections, n (%)	834 (4.4)	531 (3.5)	303 (8.1)	<0.001
Clinical manifestations of HIV, n (%)	872 (4.6)	619 (4.1)	253 (6.8)	<0.001
WHO stage, n (%)				<0.001
Ⅰ	14823 (78.4)	12288 (80.9)	2535 (68.1)	
Ⅱ	2031 (10.7)	1470 (9.7)	561 (15.1)	
Ⅲ	965 (5.1)	615 (4.0)	350 (9.4)	
Ⅳ	1092 (5.8)	813 (5.4)	279 (7.5)	
Delayed ART initiation, n (%)	9162 (48.4)	7031 (46.3)	2131 (57.2)	<0.001
Tuberculosis, n (%)	150 (0.8)	90 (0.6)	60 (1.6)	<0.001
HBV or HCV seropositive, n (%)	1040 (5.5)	902 (5.9)	138 (3.7)	<0.001
Anemia, n (%)				<0.001
Without	14143 (74.8)	11276 (74.3)	2867 (77.0)	
With	2065 (10.9)	1485 (9.8)	580 (15.6)	
Missing	2703 (14.3)	2425 (16.0)	278 (7.5)	
Fibrosis, n (%)				<0.001
Without	13487 (71.3)	10709 (70.5)	2778 (74.6)	
With	2212 (11.7)	1644 (10.8)	568 (15.2)	
missing	3212 (17.0)	2833 (18.7)	379 (10.2)	
CD4 count/μL, median (IQR)	296.0 (183.0, 414.0)	305.0 (195.0, 426.0)	254.0 (134.0, 359.0)	<0.001
CD4 count/μL, n (%)				<0.001
<200	5103 (27.0)	3771 (24.8)	1332 (35.8)	
≥200 to <500	10558 (55.8)	8600 (56.6)	1958 (52.6)	
≥500	2604 (13.8)	2267 (14.9)	337 (9.0)	
missing	646 (3.4)	548 (3.6)	98 (2.6)	
WBC, L, median (IQR)	5.4 (4.4, 6.6)	5.4 (4.5, 6.6)	5.1 (4.2, 6.3)	<0.001
WBC, L, n (%)				<0.001
<4.0 × 10^9^	2801 (14.8)	2032 (13.4)	769 (20.6)	
≥4.0 × 10^9^ to <10 × 10^9^	13199 (69.8)	10572 (69.6)	2627 (70.5)	
≥10 × 10^9^	315 (1.7)	252 (1.7)	63 (1.7)	
missing	2596 (13.7)	2330 (15.3)	266 (7.1)	
FBG, mmol/L, median (IQR)	5.3 (4.9, 5.8)	5.3 (4.9, 5.8)	5.2 (4.8, 5.7)	<0.001
FBG, mmol/L, n (%)				<0.001
<5.6	10240 (54.1)	8019 (52.8)	2221 (59.6)	
≥5.6	5280 (27.9)	4304 (28.3)	976 (26.2)	
Missing	3391 (17.9)	2863 (18.9)	528 (14.2)	
eGFR, mL/min/1.73m^2^, median (IQR)	118 (108, 125)	118 (109, 125)	117 (107, 125)	<0.001
eGFR, mL/min/1.73m^2^, n (%)				<0.001
<90	690 (3.6)	519 (3.4)	171 (4.6)	
≥90	15249 (80.6)	12087 (79.6)	3162 (84.9)	
Missing	2972 (15.7)	2580 (17.0)	392 (10.5)	
TG, mol/L, median (IQR)	1.1 (0.9, 1.7)	1.1 (0.9, 1.7)	1.2 (0.9, 1.7)	0.091
TG, mol/L, n (%)				<0.001
<1.7	14342 (75.8)	11347 (74.7)	2995 (80.4)	
≥1.7	1597 (8.4)	1259 (8.3)	338 (9.1)	
Missing	2972 (15.7)	2580 (17.0)	392 (10.5)	
TC, mol/L, median (IQR)	4.0 (3.4, 4.5)	4.0 (3.4, 4.5)	3.9 (3.4, 4.5)	0.800
TC, mol/L, n (%)				0.007
<5.2	13840 (69.1)	11075 (72.9)	2765 (74.2)	
≥5.2 to <6.2	925 (4.9)	718 (4.7)	207 (5.6)	
≥6.2	178 (0.9)	140 (0.9)	38 (1.0)	
Missing	3968 (21.0)	3253 (21.4)	715 (19.2)	
ALT, U/L, median (IQR)	22 (16, 35)	22 (16, 34)	23 (16, 36)	0.019
ALT, U/L, n (%)				<0.001
<40	13068 (69.1)	10375 (68.3)	2693 (72.3)	
≥40	3116 (16.5)	2408 (15.9)	708 (19.0)	
Missing	2727 (14.4)	2403 (15.8)	324 (8.7)	
Tbil, μmol/L, median (IQR)	11.1 (8.5, 14.5)	11.2 (8.5, 14.6)	11.1 (8.4, 14.3)	0.073
Tbil, μmol/L, n (%)				<0.001
<3.4	156 (0.8)	116 (0.8)	40 (1.1)	
≥3.4 to <20.5	14613 (77.3)	11532 (75.9)	3081 (82.7)	
≥20.5	1125 (5.9)	895 (5.9)	230 (6.2)	
Missing	3017 (16.0)	2643 (17.4)	374 (10.0)	
Advanced HIV disease				<0.001
With	5707 (30.2)	4202 (27.6)	1505 (40.4)	
Without	12558 (66.4)	10436 (68.7)	2122 (57.0)	
Missing	646 (3.4)	548 (3.6)	98 (2.6)	

### Duration and modification rates of ART regimens

Mean survival duration and ART modification rates across different intervals of follow-up were presented in Table 2. The mean of initially prescribed regimen duration in the overall sample was 31.0 (95% CI: 30.8–31.1) months. LPV/r + 3TC + AZT had the lowest mean survival duration of 18.0 (95% CI: 16.7–19.2) months, while TDF + 3TC had the highest mean survival duration of 33.5 (95% CI: 33.4–33.7) months. During the first 6 months of follow-up, TDF + AZT + NVP (Nevirapine) and LPV/r + 3TC + AZT had the highest modification rates of 59.4 (95% CI: 44.5–77.7) and 57.7 (95% CI: 48.7–68.0) per 1000 PMFU, respectively, while EFV + 3TC + TDF was lowest at 8.8 (95% CI: 8.2–9.5) per 1000 PMFU. Except DTG + 3TC + TDF and TDF + 3TC, all other ART regimens exhibited the highest modification rates within the first 6 months of follow-up.

**Table 2:** Modifications rate across initially prescribed ART regimens used.

Modification rate of ART regimens per 1000 person months (95% CI)									
Duration intervals (months)	EFV + 3TC + TDF	EFV + 3TC + AZT	LPV/r + 3TC + TDF	TDF + AZT + NVP	DTG + 3TC + TDF	LPV/r + 3TC + AZT	EVG/c/FTC/TAF	TDF + 3TC	Overall
Initially prescribed ART, N (%)	13984 (73.9)	1209 (6.4)	1843 (9.7)	185 (1.0)	320 (1.7)	504 (2.7)	719 (3.8)	147 (0.8)	18911 (100.0)
≥ 0 and < 6	8.8 (8.2–9.5)	47.4 (42.1–53.1)	36.5 (32.8–40.5)	59.4 (44.5–77.7)	23.8 (17.1–32.2)	57.7 (48.7–68.0)	47.1 (40.1–55.1)	4.6 (1.2–11.7)	16.7 (15.9–17.5)
≥ 6 and < 12	2.5 (2.1–2.8)	25.3 (21.0–30.1)	16.4 (13.7–19.5)	7.8 (2.9–17.0)	23.4 (16.0–33.0)	45.1 (36.0–55.8)	25.4 (19.5–32.6)	3.7 (0.8–10.8)	6.7 (6.2–7.2)
≥ 12 and < 18	1.9 (1.6–2.3)	20.8 (16.7–25.7)	19.1 (15.9–22.7)	5.4 (1.5–13.9)	29.4 (19.4–42.8)	39.2 (29.4–51.1)	23.2 (16.9–31.1)	14.2 (5.2–30.9)	5.8 (5.3–6.3)
≥ 18 and < 24	2.2 (1.8–2.6)	18.4 (14.3–23.4)	14.9 (11.8–18.4)	4.2 (0.9–12.4)	27.0 (15.4–43.9)	38.4 (27.5–52.3)	28.4 (20.8–37.9)	0	5.3 (4.8–5.8)
≥ 24 and < 30	1.7 (1.3–2.0)	13.6 (9.9–18.2)	18.3 (14.5–22.6)	5.8 (1.6–14.8)	43.8 (26.4–68.4)	23.9 (14.6–36.9)	24.8 (17.2–34.7)	32.6 (0.8–181.8)	4.6 (4.1–5.1)
≥ 30 and < 36	1.2 (0.9–1.6)	10.1 (6.8–14.4)	17.2 (13.2–22.0)	3.0 (0.4–10.8)	27.3 (12.5–51.9)	19.8 (10.8–33.2)	30.2 (21.0–42.0)	107.7 (13.0–389.2)	3.8 (3.3–4.3)
Mean retention time (95% CI), months	33.5 (33.4–33.7)	22.3 (21.5–23.1)	25.1 (24.4–25.7)	24.4 (22.1–26.6)	25.8 (24.4–27.2)	18.0 (16.7–19.2)	23.4 (22.4–24.5)	33.7 (32.5–34.9)	31.0 (30.8–31.1)

### Modification rates on initial ART regimens by regimen type

**Fig 3:**
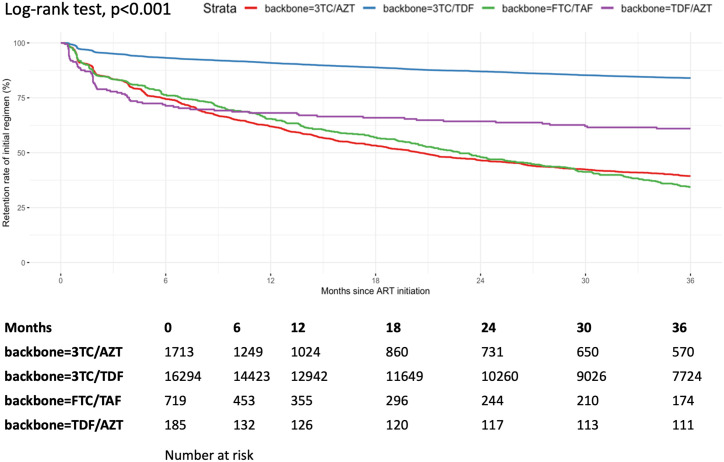
Unadjusted difference of retention rates among the initially prescribed ART backbones.

**Fig 4:**
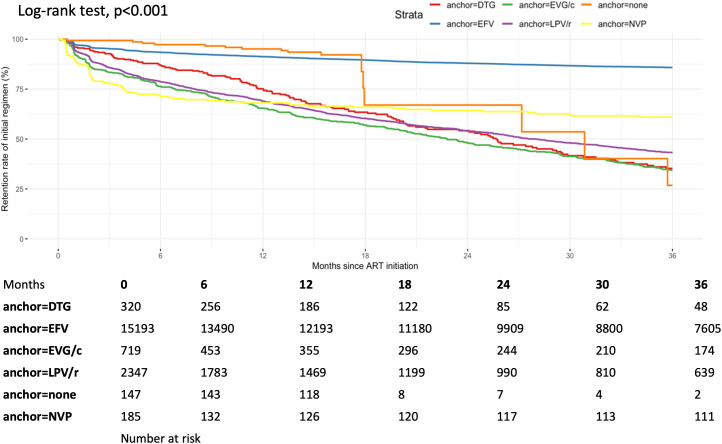
Unadjusted difference of retention rates among the initially prescribed ART anchors.

We presented unadjusted retention rates on the initially prescribed ART regimen within 36 months of follow-up, stratified by NRTI backbone (Fig 3) and anchor class (Fig 4). Retention rates differed significantly between backbones (log-rank *P* < 0.001), with TDF + 3TC achieving the highest 36-month retention and AZT + 3TC the lowest. Likewise, there was a significant difference in retention rates between anchor class (log-rank *P* < 0.001), with INSTI-based regimens (DTG and EVG/c) having the lowest risk of regimen modification compared with other groups. The unadjusted retention rates on the initially prescribed ART regimen within 36 months of follow-up were presented in S1 Fig.

### Factors associated with ART modification

The multivariable Cox proportional hazards model was presented in Table 3. Having a WBC level of <4.0 × 10⁹/L (AHR: 1.21, 95% CI: 1.11-1.31, *P* < 0.001) and missing WBC values (AHR 0.80, 95% CI: 0.69-0.93; *P* < 0.001) were associated with ART modification compared with having a WBC of 4.0-10.0 × 10⁹/L. Higher baseline WHO stage was associated with increased modification risk (stage II: AHR 1.19, 95% CI: 1.09-1.31; stage III: 1.28, 95% CI: 1.14-1.44; stage IV: 1.14, 95% CI: 1.01-1.30; *P* < 0.001, vs. stage I). Having baseline HBV or HCV coinfection was negatively associated with ART modification (AHR: 0.60, 95% CI: 0.51-0.72, *P* < 0.001). Missing TC values remain positively associated with ART modification (AHR: 1.18, 95% CI: 1.07-1.30, *P* < 0.001). Compared with EFV + 3TC + TDF, PLWH initially prescribed LPV/r + 3TC + AZT (AHR: 8.08, 95% CI: 7.16-9.12, *P* < 0.001) was associated with the highest hazard of modification. Having EFV + 3TC + AZT (AHR: 3.80, 95% CI: 3.41-4.23, *P* < 0.001), LPV/r + 3TC + TDF (AHR: 5.59, 95% CI: 5.12-6.11, *P* < 0.001), TDF + AZT + NVP (AHR: 1.94, 95% CI: 1.52-2.48, *P* < 0.001), DTG + 3TC + TDF (AHR: 6.67, 95% CI: 5.60-7.94, *P* < 0.001), EVG/c/FTC/TAF (AHR: 7.96, 95% CI: 7.09-8.95, *P* < 0.001) and TDF + 3TC (AHR: 2.19, 95% CI: 1.32-3.62, *P* < 0.001) as initially prescribed regimen were significantly and positively associated with regimen modification. Each ART initiation calendar year after 2010 was significantly associated with lower modification risk, resulting in an 83% reduction in 2020 compared with 2010 (AHR: 0.17, 95% CI: 0.10-0.31; *P* < 0.001 vs. 2010).

**Table 3:** Factors associated with ART regimen modifications.

	Adjusted Hazard Ratio, 95% Confidence Interval	*P*
Female (ref: male)	0.89 (0.78, 1.02)	0.100
WBC, L, n (%)		<0.001
≥4.0 × 10^9^ to <10 × 10^9^	Ref	
<4.0 × 10^9^	1.21 (1.11, 1.31)	
≥10 × 10^9^	1.06 (0.82, 1.36)	
missing	0.80 (0.69, 0.93)	
WHO stage		<0.001
Ⅰ	Ref	
Ⅱ	1.19 (1.09, 1.31)	
Ⅲ	1.28 (1.14, 1.44)	
Ⅳ	1.14 (1.01, 1.30)	
HBV or HCV seropositive		<0.001
Without	Ref	
With	0.60 (0.51, 0.72)	
TC, mmol/L		0.005
<5.2	Ref	
≥5.2 to <6.2	1.06 (0.92, 1.22)	
≥6.2	1.29 (0.93, 1.78)	
missing	1.18 (1.07, 1.30)	
Delayed ART initiation (ref: non-delayed ART initiation)	1.07 (1.00, 1.15)	0.052
ART regimen type		<0.001
EFV + 3TC + TDF	Ref	
EFV + 3TC + AZT	3.80 (3.41, 4.23)	
LPV/r + 3TC + TDF	5.59 (5.12, 6.11)	
TDF + AZT + NVP	1.94 (1.52, 2.48)	
DTG + 3TC + TDF	6.67 (5.60, 7.94)	
LPV/r + 3TC + AZT	8.08 (7.16, 9.12)	
EVG/c/FTC/TAF	7.96 (7.09, 8.95)	
TDF + 3TC	2.19 (1.32, 3.62)	
Year of ART initiation		<0.001
2010	Ref	
2011	0.99 (0.82, 1.18)	
2012	0.76 (0.63, 0.90)	
2013	0.62 (0.52, 0.74)	
2014	0.40 (0.33, 0.48)	
2015	0.26 (0.22, 0.32)	
2016	0.30 (0.25, 0.36)	
2017	0.39 (0.32, 0.47)	
2018	0.32 (0.26, 0.39)	
2019	0.27 (0.21, 0.34)	
2020	0.17 (0.10, 0.31)	

## Discussion

This multicenter retrospective cohort study in Beijing evaluated initially prescribed ART regimen modification among 18,911 treatment-naïve adults over 10 years. We found that nearly 1 in 5 PLWH experienced an initially prescribed ART regimen modification, with the highest modification rates occurring within the first six months for most regimens. TDF + 3TC + EFV was the predominant initial regimen with the highest durability, while LPV/r + 3TC + AZT and EVG/c/FTC/TAF were associated with higher modification rates. In the regression analyses, patients with low levels of WBC, progressed WHO clinical stage, and incomplete laboratory measures are more likely to experience treatment modification, while patients with HBV or HCV coinfection and initiated ART in later years are less likely to have treatment modification.

### Evolution of initial ART regimen choices in China

**Fig 5:**
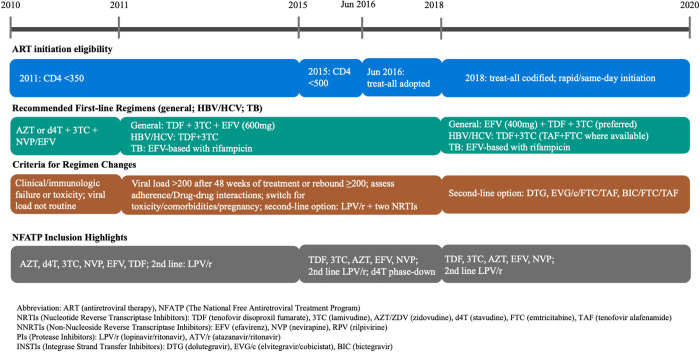
China HIV ART Policy changes: 2010-2020.

TDF + 3TC + EFV was the predominant initially prescribed regimen with a continue increase in utilization until 2018, while AZT- and PI (LPV/r)-based regimens had a decreasing trend. After 2018, the utilization of INSTIs began to rise. Prior to 2011, AZT + 3TC + EFV/NVP was the recommended first-line regimen. The 2011 edition of China’s HIV guidelines adopted the WHO recommendation of TDF + 3TC + EFV as the first choice in resource-limited settings—due to the lower hematologic and metabolic toxicity of TDF compared to AZT and NVP [27]. These changes in the national guideline were reflected in our study, where patients on AZT + 3TC + EFV changed from 28.6% in 2010 to less than 5% after 2014 and shifted to TDF + 3TC + EFV. In 2018, INSTIs (such as RAL and DTG) were officially added as options alongside EVG/c/FTC/TAF, which aligned with the increase of EVG/c/FTC/TAF and DTG + 3TC + DF from below 5% in 2018 in our study. With each guideline update (Fig 5), the risk of regimen modification for patients decreased. In our analyses, there were approximately 20% PLWH had ART modification, which is lower than the total modification rate of 25.8% in Shanghai from 2005-2013 [11]. Regimens initiated in later years were likely benefited from updated guidelines advocating for safer and more effective regimen, thereby improving retention on initial regimens [11,27].

### Modification rate of ART regimens in real-world settings

Most modifications occurred within 6 months after ART initiation with LPV/r + 3TC + AZT and EVG/c/FTC/TAF having the highest modification risk, while TDF + 3TC + EFV was the most durable. These findings were consistent with previous studies in Shanghai and nationwide, which observed a higher rate of ART switch among patients who initiated NVP- and DTG-based regimen [3,11]. In South Africa, the results were similar with the LPV/r-based regimen having the second highest risk of regimen switch compared with the EFV-based [18]. However, this was inconsistent with findings from developed countries. A retrospective, observational study in Japan found that the switch rate constantly increased for NNRTIs (17.8–45.2%) and PIs (16.2–47.6%) from 2011 to 2016, while INSTI-based regimens maintained a low switch rate (2.3–7.6%) [28]. Similarly, a multicenter cohort study in the United States found that those on EVG/c/TAF/FTC (risk ratio: 0.12, 95% CI: 0.06–0.27) switched less compared to EFV/TDF/FTC [29]. This inconsistency may be partially attributed to economic factors. In China, INSTI-based were introduced relatively late and remained comparatively costly throughout most of the study period. For example, DTG was only officially made available by the NFATP in 2021, and EVG/c/FTC/TAF has not yet been included [3]. As a result, the costs of the INSTI-based regimen typically fell under commercial health insurance, which required non-trivial patient copayments. Therefore, after achieving clinical stability following initiation of INSTI-based regimens, a greater proportion of patients may have switched to the NFATP-sponsored treatment regimens [3]. In contrast, in high-income settings, comparative health-economic analyses consistently demonstrate that INSTI-based single-tablet regimens are associated with lower total healthcare costs and higher cost-effectiveness compared to other ART regimens [30]. This cost advantage is largely driven by reduced pharmacy-related expenses and broad formulary coverage of INSTIs in these regions [30]. Consequently, cost-driven switching away from INSTI-based regimens was less prevalent in high-income settings than in China during the study period.

For LPV/r, our findings were consistent with a study conducted in Guangxi, China, which showed higher dropout rates compared to AZT-based and TDF-based regimens, likely due to the highest incidence of gastrointestinal reactions observed in those on LPV/r compared with other regimens [31]. Previous findings in Europe also indicated higher discontinuation rates for those on LPV/r compared with NVP due to toxicity or patient choice [32]. Since most modifications clustered in the first 6 months, prioritizing early follow-up (1, 3, 6 months after ART initiation) with clinical monitoring and ART adherence support is crucial to enhance the durability of the initially prescribed regimen for hospitals in China. Future research is required to evaluate the cost-effectiveness of the INSTI-based regimen in China and policies should advocate to decrease the pharmacy cost of this regimen.

### Association of baseline clinical characteristics on ART modification

Our study also found that patients with progressed WHO clinical stages at baseline ART initiation and early years of ART initiation were significantly more likely to have treatment modification. This was consistent with studies in Asmara, Eritrea, and Cape Town, South Africa, that found patients in WHO clinical stages 2, 3, and 4 at baseline were more likely to switch their ART compared to those in stage 1 [20,33]. Similarly, a retrospective study in Shanghai found that having low baseline CD4 counts was associated with high rates ART modification [11], and a global cohort study observed that children with severe baseline immunodeficiency syndrome were 1.40 times more likely to transition to second-line treatment [16]. Furthermore, another study reported significant declines in prevalence of baseline advanced HIV disease and in median time from diagnosis to treatment (from 91 days in 2011–14 days in 2018) [3] following the implementation of 2016 “treat all” policy in China [34], which might explain the annual decrease in modification risk in our study. However, diagnostic gaps persists: an estimated 360,000 people remained undiagnosed in 2018 [35], and the testing coverage among PLWH was only 84.3% in 2023 nationwide [36]. Clinical laboratory indicators also suggested persistent delay with a mean of 5.3-6.3 years from HIV infection to diagnosis reported by previous studies in China [5,37]. Together, these patterns mean that many PLWH still initiate care with baseline advanced HIV disease [21] or unrecognized comorbidities even as programs promote rapid ART initiation—circumstances that directly shape initial regimen choice and durability [11,18].

Instead of excluding people with missing baseline clinical indicators, we retained these cases to assess the effect of inadequate monitoring on treatment durability. Reasons for incomplete laboratory measure might include fear of venipuncture, concerns about privacy/disclosure and stigma, and clinic time/location inconvenience [38,39]. Our findings were similar with a previous global cohort study showing that inadequate monitoring served as a risk factor for ART regimen switches [16], as it likely reflected patients’ neglect of their health and might be linked to poor adherence or other behaviors that prompt adverse treatment outcomes. In China, NFATP has provided free first-line ART along with routine CD4 testing, viral load monitoring, HBV and HCV, as well as basic hematology and biochemistry panels (WBC, hemoglobin, liver and renal function tests) at baseline assessment [40]. However, diagnosis and treatment of opportunistic infections (e.g., antifungal therapy, hospitalization costs, tuberculosis treatment beyond first-line drugs) have not been fully covered and often require out-of-pocket contributions or reimbursement through general health insurance schemes rather than NFATP [41,42]. Patients with lower income or lack of insurance might choose not to have out-of-pocket opportunistic infections treatment, therefore more likely to have clinical comorbidity, treatment interruption, and poor health quality of life, indirectly affecting regimen durability [41,43,44].

Together, these findings underscore the importance of early ART initiation as well as continued policy attention to reduce financial barriers in comprehensive clinical monitoring and ancillary HIV care at baseline to minimize treatment modifications and avoid adverse clinical outcomes of ART for PLWH [27].

### Protective role of HBV/HCV monitoring strategies in ART durability

HBV or HCV coinfection at ART initiation was associated with a 40% lower hazard of modification in our study. This finding was likely due to well-established guidelines and enhanced monitoring recommended for PLWH with HBV or HCV coinfection. Initiating TDF-based regimens was recommended for HBV-coinfected patients in China, which can both suppress HBV replication and decrease early hematologic or hepatic toxicity [31]. Similarly, if HCV infection is present, INSTI-based regimens (most often DTG + 3TC + TDF/FTC) were recommended and clinically proven to reduce drug–drug interactions and preserve liver function [45]. This corresponds to the results in our study that participants with HBV/HCV coinfection were more likely to use a regimen with the backbone 3TC/TDF (93.8% vs.85.7% in the general population), as recommended in the guideline.

Beyond clinical guidelines, several additional mechanisms may explain this protective association. Patients with HBV or HCV coinfection routinely undergo more frequent liver function and viral load monitoring as recommended by clinics, allowing for the timely detection of subclinical toxicity or viral reactivation and thereby reducing unplanned regimen modification [46]. Additionally, patient’s awareness of coinfection may lead to better adherence and engagement in care, indirectly contributing to longer regimen durability. Moreover, the clinical complexity of HBV/HCV coinfection may lead physicians to adopt a more cautious and standardized approach to regimen selection, thereby avoiding premature or unnecessary modifications [47]. Therefore, these results underscore the value of tailored regimen selection and intensified monitoring protocols, suggesting that extending similar strategies to all PLWH could further enhance ART durability and treatment outcomes.

### Limitations

This study has several limitations. First, we did not specifically collect information regarding side effects or other self-reported reasons for regimen modification experienced by patients. However, we analyzed the modification among different regimens and found significant differences across. Also, our study focused on baseline characteristics associated with ART modification and consisted of treatment-naïve PLWH, and the detailed reasons of ART modification, such as drug resistance, should be collected and evaluated in future studies. In addition, the high missingness in some clinical laboratory measures may have affected the interpretation of risks associated with certain clinical indicators in the regression models. Furthermore, the absence of patient-level socioeconomic data limits our ability to evaluate the effect of income on treatment modification. Finally, the results of this study may not be generalizable to other countries or contexts, as data were collected in a single large city in China. However, the large sample size provides constructive recommendations.

## Conclusions

In this retrospective cohort from 2010 to 2020, nearly one in five of treatment-naïve PLWH have initially been prescribed ART modification, with most modifications occurring in the first six months. TDF + 3TC + EFV demonstrated the greatest durability, whereas AZT- and PI-based regimens (particularly LPV/r + 3TC + AZT) were more likely to undergo early modification. Having a low level of WBC and a progressed WHO clinical and incomplete laboratory evaluation significantly increased the risk of modification, while HBV/HCV coinfection and later ART initiation years had a protective effect. These findings underscored the need for early ART initiation, comprehensive pretreatment screening, and enhanced early monitoring—especially during the first six months—to optimize regimen selection and minimize unnecessary modification.

## Supporting information

S1 Fig Unadjusted difference of retention probability among the initially prescribed ART regimen. (TIFF)

S1 Table Initially prescribed ART regimen stratified by HBV/HCV coinfection. (DOCX)
