# Supplementary figures and images for: Incidence of antiretroviral therapy regimen modification and associated factors among People Living with HIV in Beijing, China
Source: PLOS Glob Public Health. 2025 Nov 3;5(11):e0005319. doi: 10.1371/journal.pgph.0005319 (PMC12582487; doi:10.1371/journal.pgph.0005319)

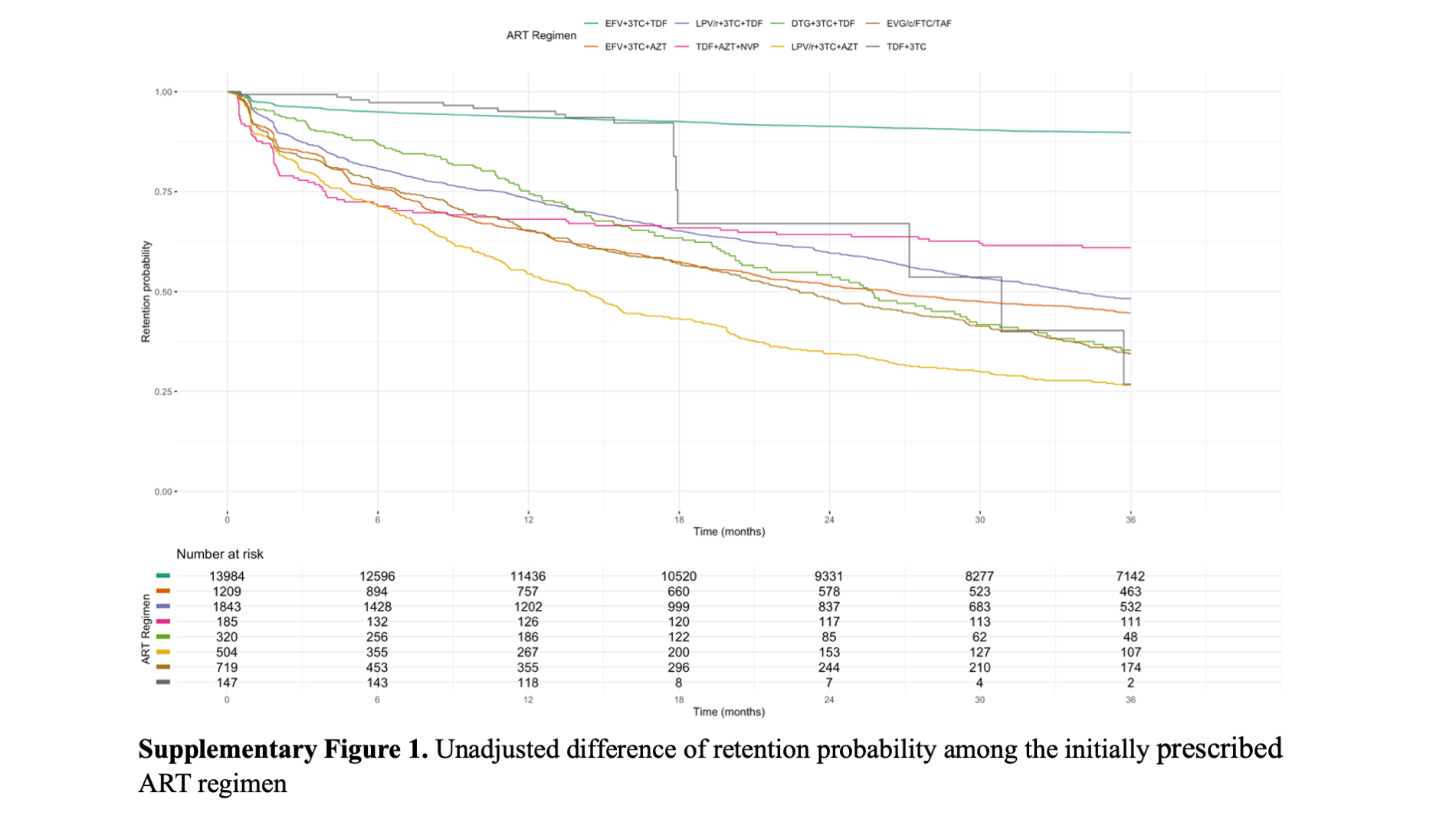

Supplement: S1 Fig — (TIFF) [file pgph.0005319.s001.tiff]
