# Supplementary material for: Incidence of antiretroviral therapy regimen modification and associated factors among People Living with HIV in Beijing, China
Source: PLOS Glob Public Health. 2025 Nov 3;5(11):e0005319. doi: 10.1371/journal.pgph.0005319 (PMC12582487; doi:10.1371/journal.pgph.0005319)
Supplement: S1 Table — (DOCX) [file pgph.0005319.s002.docx]

S1 Table. Initially prescribed ART regimen stratified by HBV/HCV coinfection

|  | level | Overall | Without HBV/HCV coinfection | With HBV/HCV coinfection | p |
| --- | --- | --- | --- | --- | --- |
| n |  | 18911 | 17871 | 1040 |  |
| backbone (%) | 3TC/AZT | 1713 ( 9.1) | 1682 ( 9.4) | 31 ( 3.0) | <0.001 |
|  | 3TC/TDF | 16294 (86.2) | 15318 (85.7) | 976 (93.8) |  |
|  | FTC/TAF | 719 ( 3.8) | 686 ( 3.8) | 33 ( 3.2) |  |
|  | TDF/AZT | 185 ( 1.0) | 185 ( 1.0) | 0 ( 0.0) |  |
| anchor (%) | DTG | 320 ( 1.7) | 306 ( 1.7) | 14 ( 1.3) | 0.008 |
|  | EFV | 15193 (80.3) | 14337 (80.2) | 856 (82.3) |  |
|  | EVG/c | 719 ( 3.8) | 686 ( 3.8) | 33 ( 3.2) |  |
|  | LPV/r | 2347 (12.4) | 2214 (12.4) | 133 (12.8) |  |
|  | none | 147 ( 0.8) | 143 ( 0.8) | 4 ( 0.4) |  |
|  | NVP | 185 ( 1.0) | 185 ( 1.0) | 0 ( 0.0) |  |
